# Comparison of lipidome profiles of *Caenorhabditis elegans*—results from an inter-laboratory ring trial

**DOI:** 10.1007/s11306-021-01775-6

**Published:** 2021-02-17

**Authors:** Britta Spanier, Anne Laurençon, Anna Weiser, Nathalie Pujol, Shizue Omi, Aiko Barsch, Ansgar Korf, Sven W. Meyer, Jonathan J. Ewbank, Francesca Paladino, Steve Garvis, Hugo Aguilaniu, Michael Witting

**Affiliations:** 1 Chair of Metabolic Programming, Technische Universität München, Gregor-Mendel-Straße 2, 85354 Freising, Germany; 2 UMR5242, Ecole Normale Supérieure de Lyon, Centre National de la Recherche Scientifique, Université de Lyon, Lyon, France; 3 Turing Center for Living Systems, Aix Marseille Univ, CNRS, INSERM, CIML, Marseille, France; 4 Bruker Daltonics, Fahrenheitstr. 4, 28359 Bremen, Germany; 5 Laboratoire de Biologie Moléculaire de la Cellule UMR5239 CNRS/ENS Lyon/UCBL/HCL Ecole Normale Supérieure de Lyon 46, allée d’Italie, 69364 Lyon cedex 07, France; 6 Instituto Serrapilheira, Rua Dias Ferreira 78, Leblon, Rio de Janeiro Brazil; 7 Research Unit Analytical BioGeoChemistry, Helmholtz Zentrum München, German Research Center for Environmental Health, Ingolstaedter Landstrasse 1, 85764 Neuherberg, Germany; 8 Metabolomics and Proteomics Core, Helmholtz Zentrum München, German Research Center for Environmental Health, Ingolstaedter Landstrasse 1, 85764 Neuherberg, Germany; 9 Chair of Analytical Food Chemistry, Technische Universität München, Alte Akademie 10, 85354 Freising-Weihenstephan, Germany

**Keywords:** *Caenorhabditis elegans*, Lipidomics, Lipid profiling, UHPLC-QToF-MS, Laboratory comparison

## Abstract

**Introduction:**

Lipidomic profiling allows 100s if not 1000s of lipids in a sample to be detected and quantified. Modern lipidomics techniques are ultra-sensitive assays that enable the discovery of novel biomarkers in a variety of fields and provide new insight in mechanistic investigations. Despite much progress in lipidomics, there remains, as for all high throughput “omics” strategies, the need to develop strategies to standardize and integrate quality control into studies in order to enhance robustness, reproducibility, and usability of studies within specific fields and beyond.

**Objectives:**

We aimed to understand how much results from lipid profiling in the model organism *Caenorhabditis elegans* are influenced by different culture conditions in different laboratories.

**Methods:**

In this work we have undertaken an inter-laboratory study, comparing the lipid profiles of N2 wild type *C*.* elegans* and *daf-2*(*e1370*) mutants lacking a functional insulin receptor. Sample were collected from worms grown in four separate laboratories under standardized growth conditions. We used an UPLC-UHR-ToF–MS system allowing chromatographic separation before MS analysis.

**Results:**

We found common qualitative changes in several marker lipids in samples from the individual laboratories. On the other hand, even in this controlled experimental system, the exact fold-changes for each marker varied between laboratories.

**Conclusion:**

Our results thus reveal a serious limitation to the reproducibility of current lipid profiling experiments and reveal challenges to the integration of such data from different laboratories.

**Supplementary Information:**

The online version contains supplementary material available at 10.1007/s11306-021-01775-6.

## Introduction

The comprehensive analysis of lipids, referred to as lipidomics or lipid profiling is becoming more frequently applied to different research topics in fundamental and applied sciences. The ultimate goal is to describe comprehensively changes in lipids and lipid profiles, ideally in a quantitative manner, and relate these changes to pathophysiological states (Wenk 2005). Mass spectrometry (MS) is the premier tool to achieve this goal and can be used with or without chromatographic separation (Züllig and Köfeler 2020). The latter is referred to as shotgun lipidomics and uses defined MS/MS experiments to identify lipids based on their fragmentation pattern and quantify them. The method can measure 100s to 1000s of lipids, but differentiation between isomeric lipid species is impossible with this technique. Additionally, due to the direct infusion approach, low abundance lipids can be suppressed by higher abundance species. In such cases, chromatographic separation should be employed. Separation of lipids is achieved either by using hydrophilic interaction liquid chromatography (HILIC) based on lipid classes (head groups) or reversed phase separation (RP) based on hydrophobicity (Züllig and Köfeler 2020).

Reproducibility is an important aspect for a successful application of lipid profiling. Analytical methodologies and approaches for lipid analysis typically show high reproducibility within a single setup or method. Comparability of lipid levels between different laboratories is, however, an important issue. Since different instruments may have other responses to the same lipids, and thus different results might be reported, comparison of these “relative” measures are difficult to undertake and integrate. Currently quantitative comparisons are only possible when results are reported as absolute concentrations, e.g. µmol/L or mg/L. In addition to the technical aspects, results may not be directly comparable between laboratories because of differences in experimental protocol, biological material, culture conditions or sample handling. In order to begin addressing these issues, a ring trial was conducted to establish reference values for lipids in the NIST SRM 1950 plasma sample (Bowden et al. 2017). This was of limited scope, however, as absolute quantification is only possible for known lipid species, for which reference standards and suitable internal standards are available. Therefore, data integration between laboratories, which perform nontargeted profiling without absolute quantification, is an important issue. For example, Izumi et al*.* compared the fold-change between two cell lines across 12 different laboratories. Their results suggested that relative values obtained from different analytical setups can be combined when the same sample was analyzed (Izumi et al. 2019). Similarly, Triebl et al. found that the use of shared reference materials beyond in-house quality controls (QCs) can even further improve the quality of data from shared studies and measurements (Triebl et al. 2020). Beside the analytical performance, pre-analytical factors like sample collection and preparation influence the outcome of studies (Burla et al. 2018; Züllig et al. 2020). In the case of microorganisms, cell cultures or model organisms, for example, the cultivation or feeding conditions influence the metabolome and lipidome.

*Caenorhabditis elegans* is one of the premier model organisms in biomedical research and is employed in basic and applied research to study aspects of development, ageing, host-microbe interactions, among others. This small nematode has been used to study lipid metabolism and especially fatty acid biosynthesis and the role of lipids in development and immunity (Kniazeva et al. 2008; Lee et al. 2010; Watts and Browse 2000, 2002; Watts and Ristow 2017). Despite this, lipidomics has only recently become part of the *C. elegans* toolbox. Lipidomic analysis is very well suited to derive better understanding of the different regulative processes as well as the role of specific lipid species in the biology of *C. elegans*. For example Gao et al*.* performed mass spectrometric analysis of lipids in order to profile the development of *C. elegans* as well as effects of feeding with different bacteria (Gao et al. 2017). Other studies have analyzed sphingolipids using shotgun or LC based lipidomics approaches (Hänel et al. 2019; Hannich et al. 2017; Mosbech et al. 2013).

Although clearly a very important step which might influence lipid metabolism in the nematode, and hence our understanding, the pre-analytical influences of cultivation are often overlooked. *C. elegans* is typically raised on a lawn of the bacteria *Escherichia coli* OP50, an uracil auxotrophic strain, following protocols developed by Sydney Brenner (Brenner 1974). This includes the use of the standard culture medium called “Nematode Growth Medium” (NGM), consisting of peptone, sodium chloride, agar, cholesterol, magnesium sulfate and potassium phosphate buffer. Peptone represents a product with variable composition that can differ between vendors. Furthermore, *E. coli* OP50 is typically grown overnight in a rich bacterial growth medium like LB broth, again based on products of high variability (tryptone & yeast extract). It has been shown that different feeding conditions can have a profound effect on the metabolome and lipidome content (Davies et al. 2012; Gao et al. 2017; Szeto et al. 2011), and that differences in the bacteria used for feeding can influence the fatty acid profile of *C. elegans* (Brooks et al. 2009). Therefore, feeding and culture conditions have a direct influence on the composition of the *C. elegans* lipidome and for this reason, likely greatly influence the outcome of lipidomics studies.

We investigated the comparability of lipid profiles from *C. elegans* cultured in different laboratories, following the “standard” protocol from Sydney Brenner (1974). We compared the wild type N2 and long-lived *daf-2(e1370)* strains. Our results reveal that while changes in several marker lipids are comparable between the individual laboratories, the exact fold changes are specific to each laboratory.

## Material and methods

### Culture of *Caenorhabditis elegans*

Fresh aliquots of *C. elegans* wild type N2 Bristol and *daf-2(e1370)* strains were ordered from the *Caenorhabditis elegans* Genetics Center (CGC), and stocks from the same plate were sent to all participating laboratories. Experiments were performed within a few weeks after arrival.

*C. elegans* were cultured on nematode growth medium (NGM) agar plates using *E. coli* OP50 as sole food source in the four different laboratories participating in this trial. After age synchronization by bleaching, the worms were grown until the first day of adulthood, harvested and washed. Each culture replicate contained approximately 5000 adult worms. Samples were snap-frozen in liquid nitrogen and stored at − 80 °C until extraction. The exact protocols from each laboratory can be found in the SI.

### Lipid extraction

Lipids were extracted by a modified version of a MTBE extraction originally developed by Matyash et al. (2008). Briefly, nematodes were suspended in 500 µL methanol and homogenized in a Precellys Bead Beating system. Subsequently, the samples were transferred to 4 mL glass vials. After addition of 1.7 mL MTBE the samples were vortexed and incubated for 60 min at room temperature. 420 µL water was added to induce phase separation. Samples were centrifuged at RCF of 15,294 × *g* and 4 °C for 15 min. The upper organic phase was transferred to fresh 4 mL glass vials and the lower phase was re-extracted with additional 650 µL MTBE. After centrifugation, the organic phases were combined and evaporated. The residue was reconstituted in 500 µL acetonitrile/isopropanol/water (65/30/5, v/v/v) and stored in 125 µL aliquots at − 80 °C until analysis.

### Lipid analysis: UPLC-UHR-ToF–MS

Lipids were analyzed as previously described (Witting et al. 2014). Briefly, lipids were separated on a Waters Acquity UPLC (Waters, Eschborn, Germany) using a Waters CORTECS UPLC C18 column (150 mm × 2.1 mm ID, 1.6 µm particle size, Waters, Eschborn Germany) and a linear gradient from 68% eluent A (40% H_2_O/60% ACN + 10 mM ammonium formate/0.1% formic acid) to 97% eluent B (10% ACN/90% iPrOH + 10 mM ammonium formate/0.1% formic acid). Mass spectrometric detection was performed using a Bruker maXis UHR-ToF–MS (Bruker Daltonic, Bermen, Germany) in positive ionization mode using data dependent acquisition to obtain MS^1^ and MS^2^ information. Every ten samples a pooled QC was injected to check performance of the UPLC-UHR-ToF–MS system and the results were used for normalization. Additional runs of QC samples in negative mode were used of lipid identification.

### Data preprocessing and statistical analysis: UPLC-UHR-ToF–MS

Raw data from the UPLC-UHR-ToF–MS was processed with Genedata Expressionist for MS 13.5 (Genedata AG, Basel, Switzerland). Preprocessing steps included noise subtraction, m/z recalibration, chromatographic alignment and peak detection and grouping. Data was exported into the Genedata Expressionist for MS 13.5 Analyst statistical analysis software and as .xlsx for further investigation.

Maximum peak intensities were used for statistical analysis and the data was normalized to the protein content of the sample. Intensity drift normalization based on QC samples was used to normalize for the acquisition sequence (Wang et al. 2013). Wild type N2 and *daf-2(e1370) C. elegans* samples were compared based on a Welch test. Results from statistical analysis were exported to .xlsx-files for further comparison between the different laboratories.

### Lipid analysis: UHPLC-TIMS-ToF–MS

A small subset of the lipid extracts was measured on a Bruker timsTOF Pro. Lipid separation was achieved on a Bruker intensity C18 column (100 mm × 2.1 mm, 1.9 µm particle size) in an Elute UHPLC (both from Bruker Daltonics, Bremen, Germany). Eluents were identical to above. A multistep gradient was used for elution: 0 min 40% B, 2 min 43% B, 2.1 min 50% B, 12 min 54% B, 12.1 min 70% B, 18 min 99% B, 18.1 min 40% B, 20 min 40% B. Column temperature was set to 55 °C and flow rate was 0.4 mL/min. Detection was carried out in positive mode using PASEF acquisition mode. The overall acquisition cycle of 0.32 s comprised one full TIMS-MS scan and two PASEF MS/MS scans with a ramp time of 100 ms and the mobility range from 0.55 to 1.9 Vs cm^−2^. The measured mass range comprised m/z 100–1350 and for fragmentation only the range of m/z 300–1350 was considered. Low-abundance precursor ions with an intensity above a threshold of 100 counts were repeatedly scheduled for fragmentation with a collision energy of 30 eV until a target value of 4000 counts was reached and then dynamically excluded for 0.1 min. For acquisition, an ion charge control setting of 5 million counts was applied. The ion mobility dimension was calibrated with a linear function using the ions derived from the ESI LC/MS tuning mix (Agilent) [m/z, 1/K_0_: (322.0481, 0.7319 Vs cm^−2^), (622.0289, 0.9848 Vs cm^−2^), (922.0097, 1.1896 Vs cm^−2^)].

### Data preprocessing and statistical analysis: UHPLC-TIMS-ToF–MS

MetaboScape® 2021 (Bruker Daltonics, Bremen, Germany) was used to process the 4-dimensional (retention time, exact mass, mobility, intensity) raw data files. The time aligned region complete feature extraction algorithm (T-ReX® 4D) extracted and aligned the features in a retention time range from 0.8 to 18 min and a mass range from 300 to 1200 m/z with an intensity of higher than 3000 and a minimum peak size in 4D space of 150 points across the sample intensity. For automatic recursive feature extraction with a lowered threshold of 100 points across the sample intensity the features needed to be present in 3 out of 21 samples. Features were excluded if not present after recursive extraction in 4 out of 21 samples. Subsequently, extracted features were filtered for a minimum of 80% presence per defined sample type (i.e. *daf-2(e1370)*, N2 and QC). The algorithm assigned averaged PASEF MS/MS spectra to the extracted features. Masses were automatically recalibrated using sodium formate clusters infused at the end of each chromatogram via a six-port valve. For ion deconvolution, [M + H]^+^ was assigned as primary ion, [M + NH_4_]^+^, [M + K]^+^, [M + Na]^+^ as seed ions and [M + H-H_2_O]^+^ as common ion. For MS/MS spectral library matching using the open source LipidBlast library (Kind et al. 2013, 2014; Ma et al. 2015; Tsugawa et al. 2020, 2017) the following maximum tolerances were applied for matching: 5.0 ppm precursor mass, isotopic fit (mSigma) 200, MS/MS score 500, 2% CCS deviation.

## Results and discussion

### Overview of the data set

Lipidomics is rapidly advancing in *C. elegans* research and is being applied in many different scientific areas. Major factors influencing the composition of nematode’s metabolome and lipidome are the culture conditions and food source (Gao et al. 2017; Szeto et al. 2011). Amino and fatty acid content varies between bacterial strains, and therefore can also influence the composition of the *C. elegans* lipidome (Brooks et al. 2009). Although the protocol for *C. elegans* culture is “standardized”, several factors can influence the outcome of metabolomics and lipidomics studies. Here we focused on the lipid profile comparison of N2 wild type and *daf-2(e1370) C. elegans* strains, the later lacking a functional insulin receptor, cultivated and harvested in four different laboratories. To avoid variances based on differences in genetic backgrounds, fresh N2 and *daf-2(e1370)* strains were ordered from the CGC from the same stock and shipped to the individual laboratories. Each laboratory produced 5 culture replicates for each genotype and harvested nematodes as young adults grown at the same temperature. The four different laboratories cultivated *C. elegans* according to the protocol established by Sydney Brenner (1974). One laboratory performed two complete independent experiments, which represent biological replicates. All samples were sent to a central laboratory where they were extracted at the same time using the same batch of solvent following the same protocol. Lipidomic analysis were performed centrally in the same laboratory. The goal of this study was to identify changes in the lipidome between N2 and *daf-2(e1370)*, but more importantly to compare the outcomes between different laboratories and the reproducibility of “biomarkers”.

**Fig. 1:**
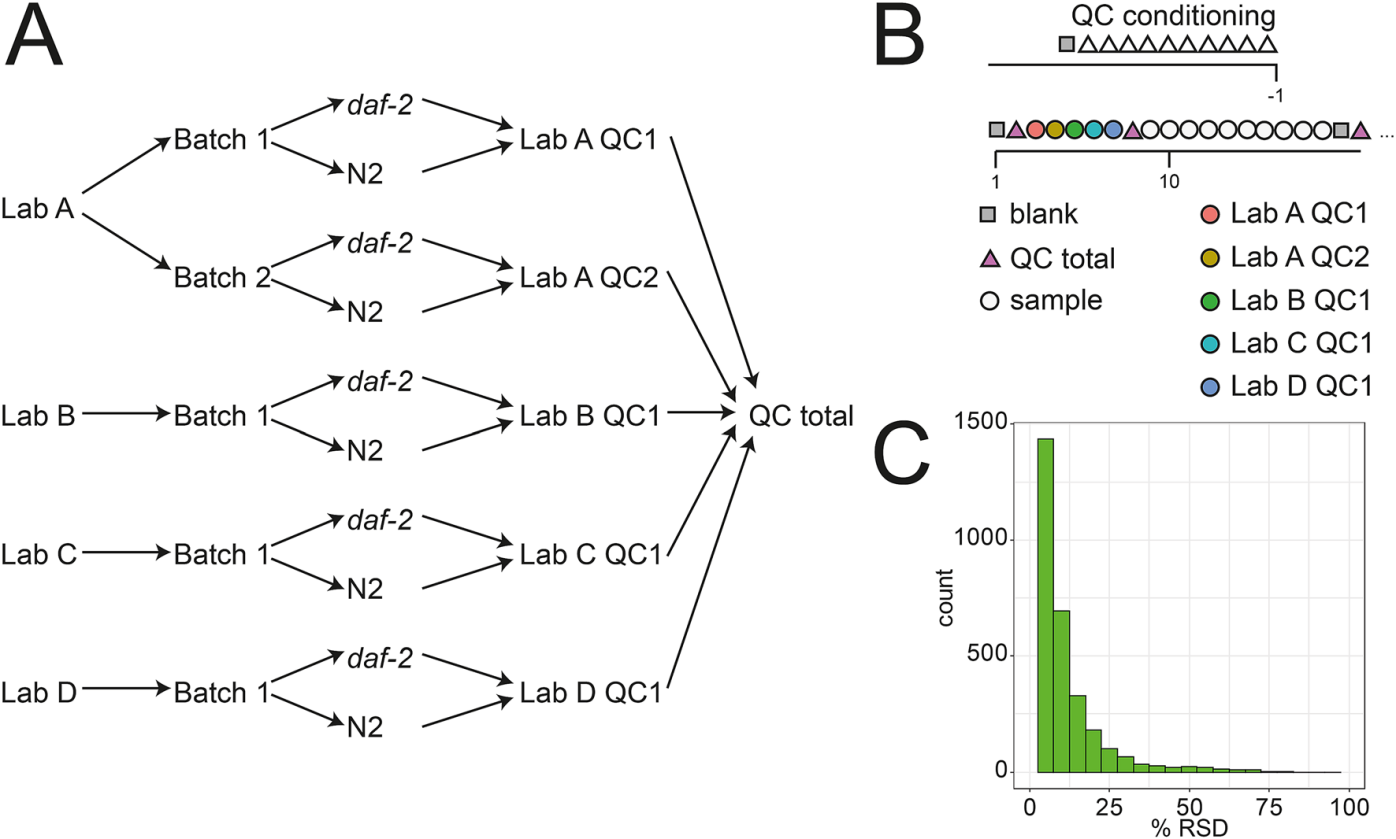
**a** Quality control (QC) scheme applied in this study. An individual QC sample was prepared by pooling aliquots from all samples from each laboratory and batch. A total QC sample was prepared by pooling aliquots from these individual QC samples. **b** Injection scheme of QC samples. Every ten samples a blank and a QC sample was injected. At the beginning and at every second QC block all individual laboratory QCs were injected. **c** Histogram of relative standard deviation (RSD) of the total QC. Features with a RSD below 30% were used for all further investigations

In order to estimate the analytical and biological variation, an extensive quality control (QC) scheme was developed (Fig. 1a, b). The total QC across all laboratories was used for intensity drift normalization (Wang et al. 2013). This total QC was based upon on pooling all samples from the entire study. Each individual sample was normalized to its protein content. After m/z recalibration, RT alignment, peak finding and isotopic clustering, a total of 4191 lipid clusters were detected across all samples. Out of these 4191, 3218 were detected in more than 80% of the QC total samples and had a relative standard deviation (RSD) < 30%. Figure 1c shows the distribution of RSDs in total QC samples.

Next, we evaluated the number of lipid clusters detected for samples from each laboratory. To be included, the feature needed to be detected individually in 80% of samples from the culture replicates of each N2, *daf-2(e1370)* and the laboratory specific QC samples, which pooled all samples of one laboratory, simultaneously (SI Figure S1). Additionally, filtering based on the total QC with > 80% frequency and < 30% RSD was used. The results are summarized in Table 1. Furthermore, the total overlap of all laboratories and batches as well as the batch and laboratory specific features were counted. Using these strict conditions, 1404 lipid clusters using the additional filtering on the total QCs were detected in all laboratories. Features that met filtering conditions in only one laboratory ranged from 5 to 233, with laboratory C showing the highest number of total lipid clusters as well as the highest number of laboratory-specific clusters. As the number of detected lipids was not too heavily influenced by additional filtering on the total QC all further statistics were performed on features that passed QC filtering criteria on the total QC (> 80% frequency, < 30% RSD) since these represent the analytically most stable of the detected lipid clusters.

**Table 1:** Number of detected features and their overlap between samples from the different laboratories

Laboratory	Batch	Total	A		B	C	D
			1	2	1	1	1
A	1	1980	–	–	–	–	–
	2	1605	1585 (98.75%)	–	–	–	–
B	1	2411	1759 (72.96%)	1476 (61.22%)	–	–	–
C	1	2918	1850 (63.40%)	1499 (51.37%)	2298 (78.75%)	–	–
D	1	2798	1867 (66.73%)	1509 (53.93%)	2281 (81.52%)	2666 (95.28%)	–

Only features that were in > 80% of each sample group (N2, *daf-2(e1370)*, QC) simultaneously from both the indicated laboratories were counted. Additionally, features have to be present in more than 80% of the total QC sample and to have an RSD < 30%

We next investigated the biological variability of replicates from N2 and *daf-2(e1370)* between the different laboratories and batches. No systematic difference in the variation measured as RSD of the culture replicates between N2 and *daf-2(e1370)* in all the different laboratories was found, suggesting that variation based on the culturing between the two strains is comparable (data not shown).

### Principal component analysis

**Fig. 2:**
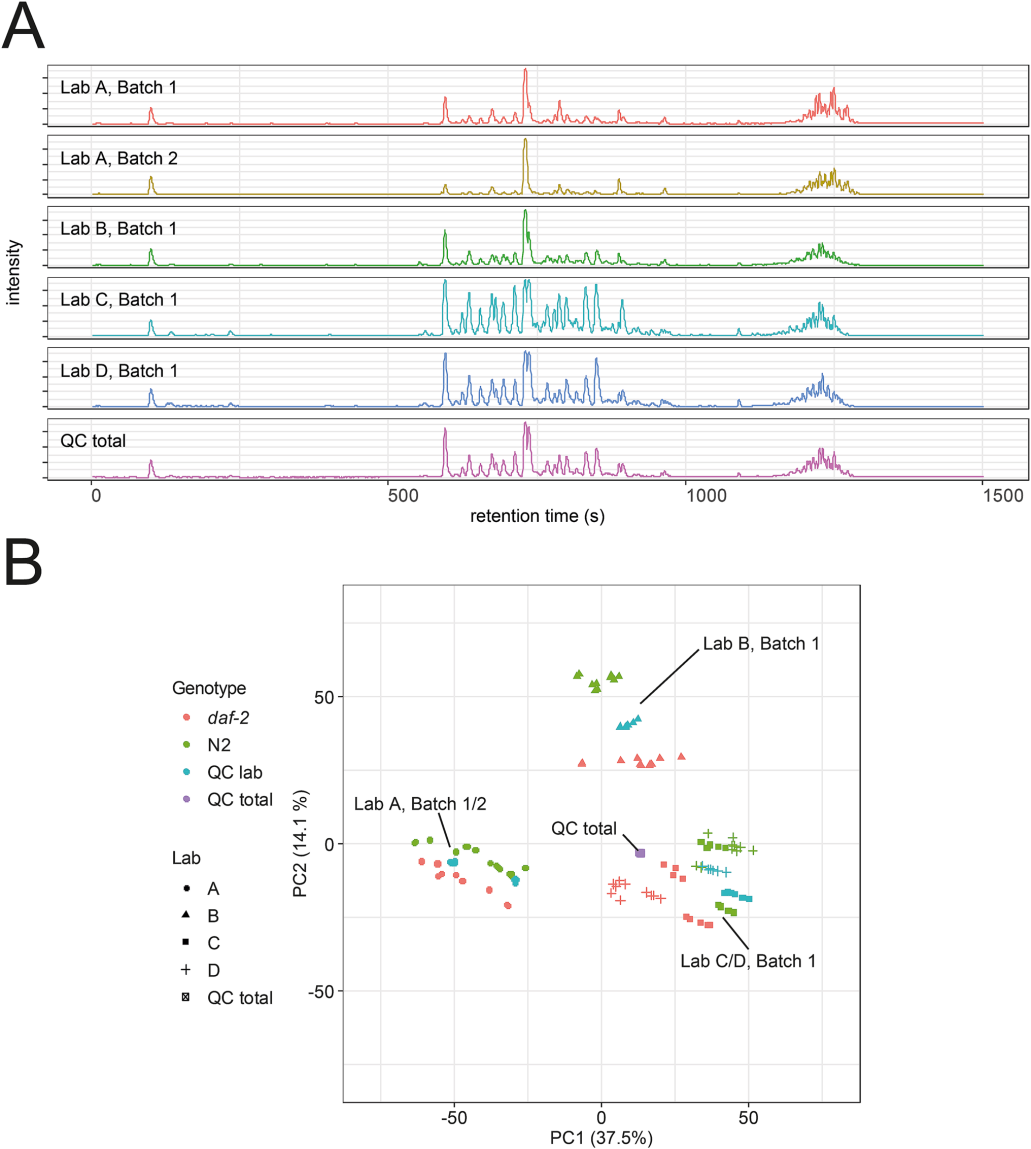
**a** Representative base peak chromatograms from laboratory-specific QC sample. **b** Principal Component Analysis (PCA) of all analyzed samples. The first principal component separated laboratory A from laboratories B, C and D. Laboratories C and D use the same chemical suppliers for the preparation of culture media and share their *E*. *coli* OP50

The first comparison discussed above indicated that the absolute number of features was influenced by the different growth conditions specific to the laboratories. Next, we wanted to examine the similarities and differences between the samples from the different laboratories. In comparing laboratory-specific QC samples, we found differences in the abundance of the different lipids that were visible in the base peak chromatograms (BPCs). To explore this variability in a systematic way, we applied principal component analysis (PCA). It is used to simplify complex high-dimensional data while retaining the trends and patterns that are inherent to the data. We performed PCA to evaluate clustering of different samples on lipid features that passed the filtering on the total QC. Along PC1 and PC2 separation was mostly based on the origin of the samples, except in the case of laboratory B for which PC2 separated the two strains in distinct groups. Three different clusters are visible. The first one is attributed to laboratory A and the two independent replicates that were produced within that laboratory. The second cluster represents laboratory B, with samples from N2 and *daf-2(e1370)* well-separated by PC2, while the third cluster represents laboratories C and D (Fig. 2b). Interestingly, laboratories C and D belong to the same institution and have the same chemical suppliers and share *E. coli* OP50 between the laboratories. For the samples from laboratories A, C and D, there was a more-or-less clear separation between N2 and *daf-2(1370)*. For each laboratory and batch, an individual PCA was performed (SI Figure S2–6). The samples from the two strains from all laboratories could be well separated along the first PC. The replicate samples from laboratory D were the most homogenous and displayed only minor variation in PC2.

### Lipids different between N2 and *daf-2*(*e1370*)

As PCA was able to separate the two genotypes in all laboratories, we were interested in discovering which lipids were driving this separation, and if they were the same in all four groups. Therefore, we performed univariate analysis comparing N2 and *daf-2(e1370)* using the Welch test. We used a p-value cut-off of 0.01 and fold-changes of either more than 2 or less than 0.5 for features significantly different between N2 and *daf-2(e1370)*. For each individual laboratory we found several hundred lipids differentially regulated between the two genotypes. To allow a robust comparison between the different laboratories we used only lipids that were detected in > 80% of all total QCs, had a RSD < 30% and were detected in > 80% of each biological group per laboratory. Interestingly, the abundance of a very small number of lipids was altered in all the laboratories with a p-value < 0.01 and a fold-change of > 2 or < 0.5. Also, notable, the number of conserved “markers” between the two independent replicate batches from laboratory A were different. In the first batch 307 lipids were found to be down-regulated in *daf-2(e1370)*, while 286 were down-regulated in the second batch, with an overlap of 166 lipids (58%). Likewise, 202 and 193 lipids were upregulated of which 121 (63%) overlapped between the two batches. The highest number of differential lipid clusters were found in laboratory D with 636 down and 523 up-regulated lipids. In total, 13 lipids were consistently down-regulated between N2 and *daf-2(e1370)* in all laboratories, while 65 were up-regulated. These lipids consistently showed low p-values, indicating that they are significantly different between the two conditions.

**Fig. 3:**
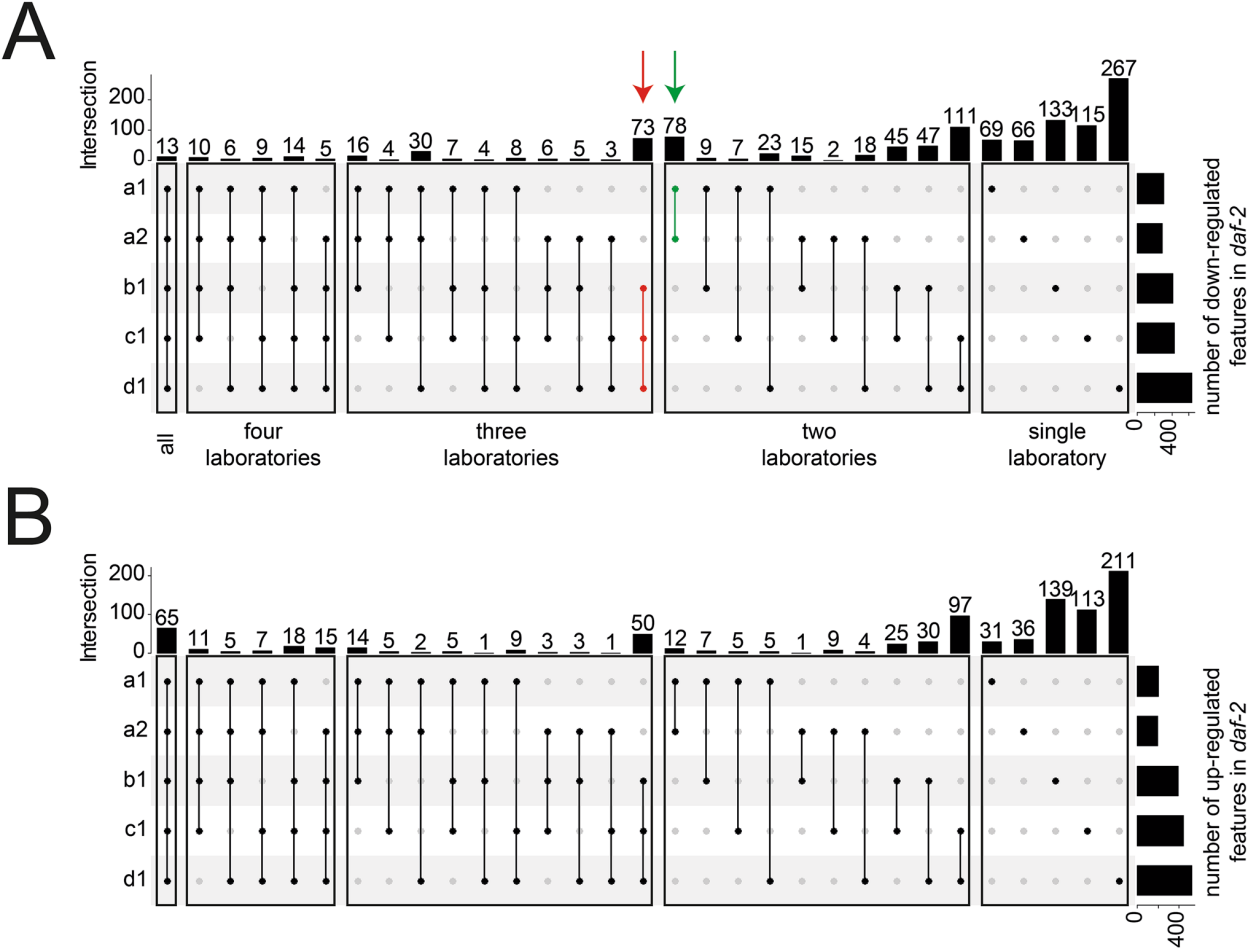
UpSet plots of the intersection between features either down- (**a**) or up-regulated (**b**). Vertical lines connect dots, which indicates which groups are compared with each other. Top bars indicate the number of distinct features in each comparison, while bars on the right show the number of total features regulated in each laboratory. **a** A relatively low overlap was observed for features down-regulated in *daf-2(e1370)*. However, two distinct cluster containing groups A1 and A2 and B1, C1, D1 are visible (green and red arrow). **b** Overlap of features up-regulated in *daf-2(e1370)* shows a higher number

Given the relative distribution of the different samples on the PCA plot, laboratories C and D would be expected to yield the highest number of overlapping marker lipids. Indeed, from all pairwise comparisons of laboratories, laboratory C and D always had the highest number of shared lipids (111 down-regulated and 97 up-regulated). Also, the overlap of markers between laboratories B and C or D was higher than any combination of laboratory A with another lab. Lastly, laboratories B, C and D also yielded the highest number of distinct features (Fig. 3).

To explore the impact of the thresholds on the recovery of conserved changed lipids between the individual laboratories, we first lowered the threshold for the fold-change to > 1 or < 1 with a p-value < 0.01. By doing so, the numbers of conserved changed lipids increased to 66 down-regulated and 121 up-regulated lipids. Lastly, we lowered the threshold of the p-value to < 0.05. Again, the numbers increased, but only slightly (98 down-regulated in *daf-2(e1370)* in all laboratories and 148 up-regulated) (SI Tables S1 and S2). These total numbers are rather low compared to the number of lipids significantly different in the individual laboratories. This indicates that differences in lipid composition between the laboratories exist and this might have similar biological consequences, but the differences do not involve the same lipid species. Most of the conserved up-regulated lipids were annotated as TGs, while the down-regulated lipids were mostly phospholipids with a high degree of unsaturation.

### Fold change differences of lipid species differentiate between the laboratories

Next, we addressed the reasons for the scarcity of overlapping markers between the laboratories. First, the fold change might be different and, in some cases, change in another direction, e.g. a certain lipid might be determined to be down-regulated in one laboratory and up-regulated in another. Secondly, as we applied an 80% cut-off for the presence of a lipid in the different groups, it was possible that a given lipid did not pass this barrier in one laboratory. To discover any additional overlapping markers, we selected lipids either up- or down-regulated in one laboratory and used the p-value and fold-change in the other laboratories for comparison. We used the lowered cut-offs for the p-values or the fold-changes as indicated above. For a better overview, p-values were divided into three different categories: p-value < 0.01, 0.01 ≤ p-value < 0.05, p-value ≥ 0.05. Likewise, we binned the fold-change into three categories. FC < 0.5, 0.5 ≤ FC ≤ 2, FC > 2.

First, we evaluated how many features showed opposite fold changes between the laboratories. Numbers for this case were low, with a maximum 7.38% of all markers that changed per laboratory. Second, we checked if the different p-values were the reason for the low overlap, using the same cut-off for the fold change, but lower p-values. Also, here the proportions were low ranging from 0.97 to 10.80%. Most of the lipid clusters showed differences in the fold change or did not pass the 80% rule. Interestingly, the 80% cut-off was the main reason for the low overlap of laboratory A samples with the others, which is consistent with the lower number of total features detected in these samples. Tables S1 and S2 summarize the different cases for lipids up- or down-regulated in *daf-2(e1370)* mutants.

**Fig. 4:**
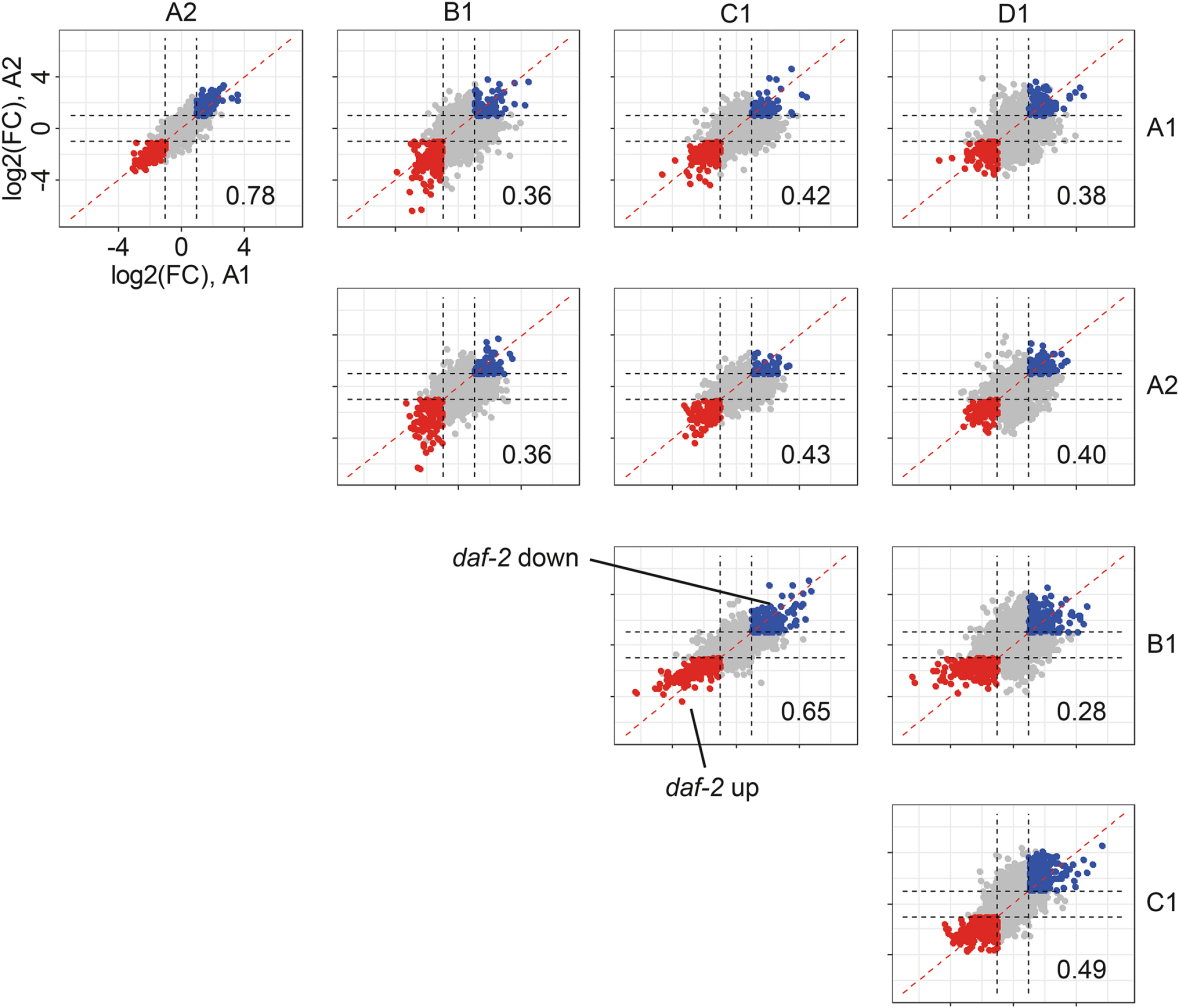
Scatter plots showing the fold changes between N2 and *daf-2(e1370)* *C*. *elegans* in the different laboratories for features commonly detected between the respective laboratories. Features up-regulated in *daf-2(e1370)* are marked red, down-regulated features are marked in blue. Numeric values indicate the correlation between the fold-changes. The higher the number the better the agreement in fold changes between the two laboratories. The highest correlation was found between the two batches from laboratory A, indicating that within a single laboratory highly reproducible results can be achieved

Based on this overview, we saw that differences in fold changes were causing the differences in the number of “markers” between the different laboratories. In order to investigate how much they were deviating from each other, we plotted the log2 of the fold change of marker lipids common between two laboratories against each other (Fig. 4). As can be seen, data points are widely scattered around the diagonal. To check how well fold changes agree between the laboratories, we performed correlation analysis. The highest correlation was observed between batch 1 and 2 from laboratory A with a Pearson correlation of 0.78, indicating that within one laboratory highly reproducible results can be obtained. Otherwise, the highest inter-laboratory correlation was for the samples from the laboratories B and C (0.65). On the other hand, consistent with the PCA analysis, the fold-change of marker lipids were poorly correlated between most of the other samples, particularly between laboratory B, D and the others (0.38, 0.4, 0.28, 0.49, for A1, A1 and C respectively).

### Lipid features not fulfilling filtering criteria

For the comparison described above, we used features that fulfilled our filtering criteria. However, the requirement to be present in > 80% of all groups (laboratory specific QC, N2 and *daf-2(e1370)* samples) might be a bias against features that have a low abundance in one biological group, but high in the other. Furthermore, features might be diluted out in the QC since it represents a pooled sample. We therefore investigated the number of features having a p-value < 0.01 and a fold-change of > 2 or < 0.5, but that are only present in one group in > 80% of all samples (e.g. if a feature is down-regulated in samples from *daf-2(e1370)* it had to be present in > 80% of N2 samples, but could be present in ≤ 80% of the *daf-2(e1370)* samples). All numbers are summarized in Table 2. Using these criteria several additional potential “markers” have been added. However, investigating these features we found that most of them were of low intensity across all analyzed samples.

**Table 2:** Number of additional “markers” when the requirement for > 80% presence in all groups is omitted in one of the statistical groups

Laboratory	Batch	*daf-2(e1370)* down-regulated > 80% N2 ≤ 80% *daf-2(e1370)*	*daf-2(e1370)* up-regulated > 80% *daf-2(1370)* ≤ 80% N2
A	1	8	21
	2	74	51
B	1	42	76
C	1	46	36
D	1	103	53

### Comparison with previously described “biomarkers” of *daf-2* knockout-mutants

After finding that the fold change determines a major part of the difference between the results from the four laboratories, we investigated if any of the several marker lipids identified are consistent with previous comparisons between N2 and *daf-2(e1370)*. Changes in the lipid profiles between N2 and *daf-2(e1370)* have been described by numerous investigations. *daf-2(e1370)* are commonly described to have a fat phenotype, with increased lipid content. This is mirrored by an elevation of triacylglycerols (TGs). Prasain et al*.* used the total ion intensity of all TGs species to quantify the differences between N2, *daf-1(m40)* and *daf-2(e1370).* The latter showed the highest amount of TGs (Prasain et al. 2015).

We estimated the total TG content in the same way by summing the intensities of all peaks putatively annotated as TGs. TGs are ionized as [M + NH_4_]^+^ adducts and occupy a distinct space in the RT-m/z plane and therefore can be easily identified and annotated. Consistent with the previously reported fat phenotype, we observed higher TG content in *daf-2(e1370)* mutants in all laboratories except for laboratory A. Batch 1 showed no significant difference between WT and *daf-2(e1370)*, while in batch 2 only a modest, but still statistically significant increase was observed. Next, individual markers were investigated. We started with features that are conserved in all laboratories. Consistently, 13 features were down-regulated in *daf-2(e1370)* in all laboratories, while 65 were up-regulated. In agreement with the previous observations of higher TGs levels, 45 of these 65 were annotated as TG. Castro et al*.* performed a metabolomic and lipidomic analysis of N2 and *daf-2(e1370)* (Castro et al. 2013). They reported an increase of TGs containing monounsaturated and branched chain fatty acids in *daf-2(e1370)*. Based on our annotations we can confirm these results. The 45 TGs were on the lower range in regard to the number of carbons in the side chains compared with all detected TGs.

**Fig. 5:**
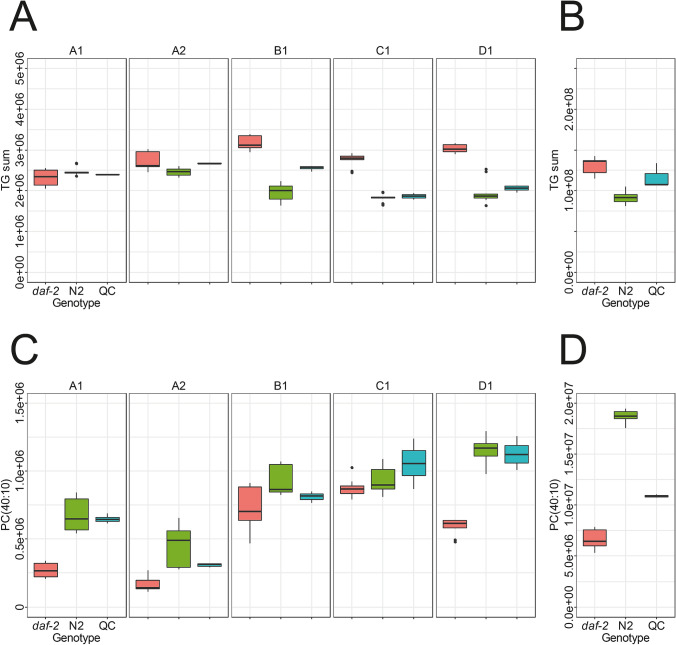
**a** Boxplot of the sum of all features annotated as TGs used as proxy for the quantity of lipid droplets from laboratories A to D. In most laboratories up-regulation of the TG content in *daf-2(e1370)* was observed. **b** Boxplot of the sum of all features annotated as TGs used a proxy for the quantity of lipid droplets from the timsTOF Pro data set. Similar to the previous results an increase in TG content in *daf-2(e1370)* samples was observed. **c** Boxplot of PC(40:10) from laboratories A to D. In all laboratories except for laboratory C a significant down-regulation was found. **d** Boxplot of PC(40:10) from the timsTOF Pro data set

Another change in profile that was identified by Castro et al*.* was the reduction of PC(20:5/20:5) in *daf-2(e1370)* (Castro et al. 2013). We identified a peak eluting at 9.9 min as PC(20:5/20:5). Consistent with previous results this peak is significantly down-regulated in *daf-2(e1370)* mutant worms, except in laboratory C, where it shows only minor changes (Fig. 5). From the list of down-regulated markers, other lipids were tentatively annotated as phospholipids with high degree of unsaturation.

### Comparison against a different lipidomics platform

Finally, we wished to determine if similar results could be obtained using a different analytical platform. A selection of samples from laboratory D were analyzed a second time using UHPLC-TIMS-ToF–MS with another column and gradient system. Lipids identified by rule based lipid annotation were further analyzed. In total 2102 features were detected and out of these 431 had a minimum of one annotation.

Similar to all the other datasets, PCA was able to separate N2 from *daf-2(e1370) C. elegans* on the first principal component (SI Figure S6). Based on the t-test functionality in MetaboScape, a comparison of N2 and *daf-2(e1370)* yielded 510 lipid features that were found to be down-regulated and 84 up-regulated. Since different chromatographic conditions, additional complementary ion mobility separation and mass spectrometric detection were used, a direct comparison is only possible for identified lipids. Focusing on the two examples previously described, we could see the same trends in the present dataset (Fig. 5).

PC(20:5/20:5) was also down-regulated in the timsTOF Pro data set. Of note, the acquired PASEF data permitted lipid annotation considering the collisional cross section value (CCS) value. The measured CCS value of 287.2 for the [M + H]^+^ of PC(20:5/20:5) matched within 0.6% to the predicted values contained in LipidBlast (Tsugawa et al. 2020; Zhou et al. 2017). The sum of features annotated as TGs was also increased in this analysis of *daf-2(e1370)* samples. From the up-regulated features, several were annotated as TGs, and consistent with previous results they were also shorter on average compared to the whole population of the TGs.

## Conclusion

Reproducibility of data is a major issue in science and requires meticulous attention to even the smallest detail. When working with biological systems, especially live organisms, the intrinsic differences between individuals can be amplified by environmental and experimental variations. A current major question relates to how scientists can determine which results obtained in different laboratories can be compared, and how best to control and correlate these data. In metabolomics and lipidomics this question is manifold, since no accepted universal protocol exists. In *C. elegans* lipidomic profiles are closely related to culture conditions of the nematodes. We therefore investigated the reproducibility of lipid profiles comparing N2 and *daf-2(e1370)* worms grown in four different laboratories.

Using lipid profiling based on UPLC-UHR-ToF–MS, two different *C. elegans* strains were compared. Based on pairwise comparison, “markers” for the difference between these strains were identified. When comparing these markers between the different laboratories, only a small overlap was identified, indicating that even for a relatively simple biological model for which culture conditions are standardized, uncontrolled variables have a strong effect on the lipid profile and the outcome of lipidomic studies. In this study we used stringent filtering conditions, e.g. we required features to be present in > 80% of each sample group (N2, *daf-2(e1370)* and QC). In order to be regarded as significantly different between the two strains, we defined p-value to be < 0.01 and a fold-change smaller than 0.5 or larger than 2. We identified differences in fold changes between N2 and *daf-2(e1370)* or the criteria of being present in minimum 80% of each group as the main variables accounting for the low data overlap between the sample groups. Despite the inter-laboratory differences, however, the biochemical changes known to characterize the *daf-2(e1370)* mutant, e.g. increased content of TGs with lower chain length of fatty acid side chains or the down-regulation of phospholipids with PUFAs, were confirmed in the present study. Thus, the major hallmarks of *daf-2(e1370)*-specific lipid profile are sufficiently robust as to be visible despite the extensive inter-laboratory variation.

Although general trends are conserved between the laboratories and consistent with previously published results, the exact fold-changes for individual lipid species are not the same. This can be attributed to the slight differences in the culture and feeding conditions since the same extraction and analytical conditions were used in the initial set of measurements. One particular example is the *E. coli* OP50 used for feeding. One laboratory used DYT-medium for the growth of bacteria, while all others used LB medium. Furthermore, two of the three laboratories used “home-made” LB medium, while one used pre-made powder for the preparation. Furthermore, the exact OD of the culture used for seeding of NGM plates is often not determined and typically simply an aliquot of an overnight culture is used. Differences in the metabolic state and therefore the composition of feeding bacteria will be reflected in differences of the *C. elegans* metabolome and lipidome. Using another analytical platform, similar findings were obtained for one selected set of samples. Interestingly, although identical samples were measured using the different methods and instrumentation, different fold-changes were found for the same identified lipids, e.g. PC(20:5/20:5). This might be attributed to the different chromatographic separation used as well as different processing software and settings.

A major question is how lipid analysis can be conducted in the future to allow cross-laboratory integration of data sets for system wide analysis. The first possibility is to generate quantitative data. This helps to remove analytical bias, but not variation due to culture conditions. This type of analysis would also be an important step towards the construction of a *C. elegans* reference lipidome. The second possibility is to standardize further and harmonize culture conditions. For example, while studying folate synthesis, Virk et al*.* found inconsistent results in life span analysis, when using standard agar. The use of high-purity agar resolved this problem (Virk et al. 2012). Another example is the use of tryptone and peptone for the culture of food bacteria and growth of *C. elegans*. The data sets of laboratories using the same chemicals clustered closer together in PCA, indicating lower differences in lipid compositions. Since both tryptone and peptone are a “natural” product with some batch-to-batch and provider variability, their replacement with a synthetic or more highly purified form could further reduce variability. An alternative to these media components might be a defined amino acid and peptide mixture, which can be produced in more standardized fashion. Lastly, the inclusion of a *C. elegans* reference sample can help improving the comparability between different laboratories and their analytical methods, following the example of the human NIST reference sample (Bowden et al. 2017; Simón-Manso et al. 2013). However, such a reference sample needs to be produced and characterized first.

While our results represent an important first step, in the future further investigations into the comparability and standardization of *C. elegans* lipidomics, and more broadly metabolomics, are required. Better comparability will enable integration of results from different laboratories and analytical setups into a comprehensive systems analysis of lipid metabolism.

## Supplementary Information

Below is the link to the electronic supplementary material.

Supplementary file1 (DOCX 839 KB)

## Data Availability

The metabolomics and metadata reported in this paper are available via Metabolights (https://www.ebi.ac.uk/metabolights/) study identifier MTBLS2016.
