# Supplementary material for: Comparison of lipidome profiles of Caenorhabditis elegans—results from an inter-laboratory ring trial
Source: Metabolomics. 2021 Feb 17;17(3):25. doi: 10.1007/s11306-021-01775-6 (PMC7886748; doi:10.1007/s11306-021-01775-6)
Supplement: Supplementary file 1 — Supplementary file1 (DOCX 839 KB) [file 11306_2021_1775_MOESM1_ESM.docx]

**Comparison of lipidome profiles of *Caenorhabditis elegans* – Results from an inter-laboratory ring trial – Supplemental Information**

Britta Spanier^1^, Anne Laurençon^2^, Anna Weiser^1^, Nathalie Pujol^3^, Shizue Omi^3^, Aiko Barsch^4^, Ansgar Korf^4^, Sven W. Meyer^4^, Jonathan J. Ewbank^3^, Francesca Paladino^5^, Steve Garvis^5^, Hugo Aguilaniu^2,6^, Michael Witting^7,8,9,^*,

1 Chair of Metabolic Programming, Technische Universität München, Gregor-Mendel-Straße 2, 85354 Freising, Germany.

^2^ Ecole Normale Supérieure de Lyon, Centre National de la Recherche Scientifique, Université de Lyon, UMR5242, Lyon, France

^3^ Aix Marseille Univ, CNRS, INSERM, CIML, Turing Center for Living Systems, Marseille, France

^4^ Bruker Daltonics, Fahrenheitstr. 4, 28359 Bremen, Germany

^5^ Laboratoire de Biologie Moléculaire de la Cellule UMR5239 CNRS/ENS Lyon/UCBL/HCL Ecole normale supérieure de Lyon 46, allée d'Italie 69364 Lyon cedex 07, France

^6^ Instituto Serrapilheira, Rua Dias Ferreira 78, Leblon, Rio de Janeiro, Brazil

^7^ Research Unit Analytical BioGeoChemistry, Helmholtz Zentrum München, German Research Center for Environmental Health, Ingolstaedter Landstrasse 1, 85764 Neuherberg, Germany

^8^ Metabolomics and Proteomics Core, Helmholtz Zentrum München, German Research Center for Environmental Health, Ingolstaedter Landstrasse 1, 85764 Neuherberg, Germany

^9^ Chair of Analytical Food Chemistry, Technische Universität München, Alte Akademie 10, D-85354 Freising-Weihenstephan, Germany

* corresponding author:

Dr. Michael Witting, michael.witting@helmholtz-muenchen.de

# Chemicals

## Buffer and culture recipes

Although the preparation of NGM normally follows the protocol of Sydney Brenner, different versions have been developed. Below the different recipes from the different laboratories are summarized. All other parts of the protocol were mostly identical, except that laboratories A and D used M9 buffer for washing of worms, while laboratory C and B used 50 mM NaCl.

### Laboratory A

#### Bacterial growth medium

Laboratory A used DYT-medium for growing food bacteria *E. coli* OP50. Dissolve 5 g NaCl, 10 g bacto yeast extract and 16 g Casein-peptone in 1 L H_2_O and autoclave. Aliquot in 50 mL bottles, autoclave again and store at 4°C.

#### Nematode Growth medium (NGM)

Dissolve 3 g NaCl, 2.5 g Casein-peptone (Roth, Germany), 17 g high-strength agar (Serva, Germany) in 1 L H_2_O and autoclave. Add 1 mL cholesterol (0.5 g in 100 mL EtOH), 0.5 mL 1M CaCl_2_, 1 mL 1M MgSO_4_, 25 mL 1 M potassium phosphate buffer (pH 6.0) and 10 mL nystatin (1g nystatin in 100 mL ammonium acetate / 96% EtOH (1:1)). 9 cm NGM plates were seeded with 50 µL of an overnight culture of *E. coli* OP50 grown in DYT-medium.

#### Bleach Solution

2 x Bleach solution was prepared by mixing 0.5 mL 12% NaOCl, 1.25 mL 5 M KOH and 23.25 mL autoclaved H2O.

#### M9 Buffer

Dissolve 3 g KH_2_PO_4_, 6 g Na_2_HPO_4_, 5 g NaCl in 1 L H_2_O. Sterilize by autoclaving. Add 1 mL of 1 M MgSO_4_ when cooled down to room temperature.

#### C. elegans culture

Large amounts of worms were grown by seeding 10 L4 larvae per plate and strain to 6 cm NGM-agar plates with *E. coli* OP50 as food source. Plates were overgrown until most of the bacteria have been fed. The starting plates were chunked into 3 pieces on three fresh 9 cm NGM-agar plates. Plates were again overgrown until most of the bacteria have been fed and areas that contain most worms were chunked and placed on 10 fresh 9 cm NGM-agar plates. Plates were again overgrown until most of the plates have been fed and worms were washed of the plates with M9 buffer and collected in 15 mL falcon tubes. After centrifugation for 2 min at 2500 rpm the supernatant was removed and worms were washed two more times with M9 buffer, whereby after the last wash worm were left in 3 mL buffer.

Worms were synchronized by bleaching by adding 3 mL 2 x Bleach solution and shaking for 3-4 minutes. Lysis of worms was checked under a microscope. When the adults started to break to release the eggs, samples were centrifuged for 2 min at 2500 rpm. Samples were washed minimum 5 times and after the last wash 7 mL M9 buffer were added and samples were incubated overnight at 20°C with gently shaking to allow a permanent oxygen supply. The next day the falcon tubes were placed in an upward position for 1-2 min to let the worm debris settle. The supernatant with the L1 larvae was transferred to a fresh tube and the L1 larvae were washed once with M9 buffer and worms collected by centrifugation for 2 min at 2500 rpm. Roughly 1000 L1 larvae were added to each of 5 fresh 9 cm NGM-agar plate with *E. coli* OP50 where the worms developed to young adults. Worms were grown at 20°C. Worms were washed of the plates with water in 15 mL falcon tubes and washed at least 5 times with water till the supernatant was clear. The pellet was transferred to a reaction tube and most of the supernatant was removed. Samples were snap-frozen in liquid nitrogen and stored at -80°C.

### Laboratory B

#### Bacterial Growth Medium

Laboratory B used LB medium for growing food bacteria (*E. coli* OP50).

#### Nematode Growth medium (NGM)

Dissolve 3 g NaCl, 2.5 g Bacto-Peptone (BectonDickinson/Difco), 20 g BactoAgar and 1 mL cholesterol (0.5 g in 100 mL EtOH) in 1 L deionized water and autoclave for 20 minutes at 121 °C. Add 1 mL of 1 M CaCl_2_, 1 mL 1 M MgSO_4_ and gently swirl before adding 25 ml of 1.25 M phosphate buffer (pH 6.0). 10 cm NGM plates were seeded with 1 mL of an overnight culture of *E. coli* OP50 grown in LB medium at 37°C.

#### Bleach solution

Bleach solution was prepared by mixing 5 mL 50mM NaCl, 3 mL Bleach and 0.5 mL NaOH 50%.

#### 50 mM NaCl

Dissolve 1.463 g NaCl in 500 mL H_2_O and autoclave.

#### C. elegans culture

Worms cultured at 20°C were chunked onto a seeded 10 cm NGM plate and grown 3-4 days until there were a lot of eggs and gravid adults on the plate. Worms and eggs were transferred to a 15 mL falcon tube with 50 mM NaCl and centrifuged for 2 min at 2000 rpm. Most of the supernatant was removed and about 10 mL of bleach added. Tubes were agitated by inverting gently approximately 2 minutes or until a decrease in the number of intact adult worms was observed. Once most of the bodies have dissolved the sample was centrifuged for 2 min at 2000 rpm. Eggs were washed three times with about 15 mL of 50 mM NaCl. After the last washing step most of supernatant was removed and about 1 mL of 50 mM NaCl were added. Eggs were hatched overnight with gentle rocking at 20°C. About 1000 worms per plate were distributed onto 2 seeded 10 cm NGM plates and cultured at 20°C for 48 hrs. Worms were then transferred to 15 mL falcon tube with 50 mM NaCl and centrifuged for 2 min at 2000 rpm. After removal of most of the supernatant the pellet was transferred to 2 seeded 10 cm NGM plates and culture at 25°C for 6 hours. Worms are transferred to 15 mL falcon with 50 mM NaCl and washed three times with 50 mM NaCl. After the last washing step, the supernatant was removed, and worms suspended in 1 mL 50 mM NaCl and transferred to a 1.5 mL Eppendorf tube. After centrifugation for 2 min at 2000 rpm the supernatant was removed and samples were snap-frozen in liquid nitrogen and stored at -80°C.

### Laboratory C

#### Bacterial Growth Medium

Laboratory C used LB medium for growing food bacteria (*E. coli* OP50).

#### Nematode Growth medium (NGM)

Dissolve 3 g NaCl, 2.5 g Bacto-Peptone (BectonDickinson/Difco) and 20 g Agar-Agar in 975 mL deionized water and autoclave for 50 minutes at 121°C. Add 1 mL of 5 mg/mL cholesterol, 1 mL of 1 M CaCl_2_, 1 mL 1 M MgSO_4_ and gently swirl before adding 25 ml of 1 M phosphate buffer pH 6. 9 cm NGM plates were seeded with 200 mL of an overnight culture of E. coli OP50 grown in LB medium at 37°C.

#### Bleach solution

Bleach solution was prepared by mixing 5.5 mL H_2_O, 2.5 mL 1M NaOH and 2 mL bleach.

#### 50 mM NaCl

Dissolve 1.463 g NaCl in 500 mL H_2_O and autoclave.

#### C. elegans culture

Worms were grown at 20°C on NGM plates and synchronized by bleaching. After bleaching, worms were washed three times with 50 mM NaCl. Eggs were deposited onto non-seeded NGM plates and incubated overnight at 20°C for hatching and to allow for synchronize population. Worms were then transferred onto seeded 9 cm NGM plates and incubated at 20°C and allowed to develop to young adult stage. Worms were washed of the plate with 50 mM NaCl and washed three times. After the last washing step, the supernatant was removed and samples were snap-frozen in liquid nitrogen and stored at -80°C.

### Laboratory D

#### Bacterial Growth Medium

Laboratory D used LB broth Lennox from BectonDickison for growing food bacteria (*E. coli* OP50).

#### Nematode Growth medium (NGM)

Dissolve 3 g NaCl, 2.5 g Bacto-Peptone (BectonDickinson/Difco) and 20 g Agar-Agar in 975 mL deionized water and autoclave. Add 1 mL of 5 mg/mL cholesterol, 1 mL of 1 M CaCl_2_, 1 mL 1 M MgSO_4_ and gently swirl before adding 25 ml of 1 M phosphate buffer pH 6. 5.5 cm NGM-agar plates were seeded with 180 µL and 9 cm NGM plates were seeded with 1 mL of an overnight culture at 37°C of *E. coli* OP50.

#### Bleach solution

Bleach solution was prepared by mixing 2 mL Clorox, 3 mL H2O and 5 mL 1 M NaOH.

#### M9 buffer

Dissolve 3 g KH_2_PO_4_, 6 g Na_2_HPO_4_, 5 g NaCl in 1 L H_2_O. Sterilize by autoclaving. Add 1 mL of 1 M MgSO_4_ when cooled down to room temperature.

#### C. elegans culture

Five adult worms were seeded on a 55mm NGM plate and cultured at 20°C for 4 days. Worms were washed off plates with M9 buffer and centrifuged for 2 min at 4200 rpm. Supernatant was removed and 9 mL of bleach solution were added. Samples were vortexed periodically until worms dissolve. Afterwards, samples were centrifuged for 2 min at 4200 rpm and 4°C and washed three times with M9. Eggs were allowed to hatch overnight by shaking in an Erlenmeyer. L1 larvae were harvested and centrifuged for 2 min at 2200 rpm. 90 mm NGM plates were seeded with 1000 worms and cultured at 20°C until young adults. Worms were washed off the plates with cold M9 and centrifuged for 2 min at 4200 rpm and 4°C and wash three times with M9. After the last washing step samples were snap-frozen in liquid nitrogen and stored at -80°C.

SI Table 1: daf-2 down regulated (binning)

|  |  |  | **fold change** | **>2** | | | **0.5 < x < 2** | | | **<0.5** | | | **not detected in each group >80%** |
| --- | --- | --- | --- | --- | --- | --- | --- | --- | --- | --- | --- | --- | --- |
| **Lab** | **Batch** | **Lab** | **p-value** | **<0.01** | **0.01 < x < 0.05** | **>0.05** | **<0.01** | **0.01 < x < 0.05** | **>0.05** | **<0.01** | **0.01 < x < 0.05** | **>0.05** |  |
|  |  |  | **Batch** |  |  |  |  |  |  |  |  |  |  |
| **A** | **1** | **A** | **2** | 166 | 4 | 0 | 24 | 3 | 8 | 0 | 0 | 0 | 105 |
|  |  | **B** | **1** | 79 | 0 | 0 | 91 | 24 | 62 | 19 | 0 | 0 | 36 |
|  |  | **C** | **1** | 72 | 2 | 0 | 98 | 27 | 75 | 14 | 0 | 0 | 24 |
|  |  | **D** | **1** | 107 | 2 | 0 | 70 | 20 | 66 | 20 | 0 | 0 | 27 |
| **A** | **2** | **A** | **1** | 166 | 6 | 0 | 63 | 20 | 28 | 0 | 0 | 0 | 4 |
|  |  | **B** | **1** | 76 | 1 | 0 | 95 | 24 | 51 | 18 | 0 | 0 | 22 |
|  |  | **C** | **1** | 52 | 1 | 0 | 98 | 31 | 78 | 16 | 0 | 0 | 11 |
|  |  | **D** | **1** | 89 | 1 | 0 | 78 | 12 | 70 | 17 | 1 | 0 | 19 |
| **B** | **1** | **A** | **1** | 79 | 10 | 2 | 40 | 40 | 68 | 3 | 1 | 0 | 176 |
|  |  | **A** | **2** | 76 | 3 | 1 | 54 | 6 | 50 | 0 | 0 | 0 | 229 |
|  |  | **C** | **1** | 173 | 7 | 0 | 99 | 17 | 82 | 1 | 0 | 0 | 40 |
|  |  | **D** | **1** | 170 | 2 | 1 | 80 | 22 | 65 | 14 | 0 | 0 | 65 |
| **C** | **1** | **A** | **1** | 72 | 11 | 6 | 27 | 20 | 31 | 3 | 0 | 0 | 282 |
|  |  | **A** | **2** | 52 | 2 | 0 | 25 | 5 | 18 | 1 | 0 | 0 | 349 |
|  |  | **B** | **1** | 173 | 0 | 1 | 72 | 10 | 45 | 1 | 0 | 0 | 150 |
|  |  | **D** | **1** | 239 | 0 | 0 | 50 | 10 | 35 | 2 | 0 | 0 | 116 |
| **D** | **1** | **A** | **1** | 107 | 18 | 8 | 37 | 30 | 54 | 5 | 1 | 0 | 393 |
|  |  | **A** | **2** | 89 | 3 | 1 | 21 | 9 | 38 | 3 | 0 | 0 | 489 |
|  |  | **B** | **1** | 170 | 3 | 2 | 154 | 40 | 112 | 29 | 1 | 0 | 142 |
|  |  | **C** | **1** | 239 | 17 | 2 | 166 | 46 | 146 | 10 | 0 | 0 | 27 |

SI Table 2: daf-2 up regulated (binning)

|  |  |  | **fold change** | **>2** | | | **0.5 < x < 2** | | | **<0.5** | | | **not detected in each group >80%** |
| --- | --- | --- | --- | --- | --- | --- | --- | --- | --- | --- | --- | --- | --- |
| **Lab** | **Batch** | **Lab** | **p-value** | **<0.01** | **0.01 < x < 0.05** | **>0.05** | **<0.01** | **0.01 < x < 0.05** | **>0.05** | **<0.01** | **0.01 < x < 0.05** | **>0.05** |  |
|  |  |  | **Batch** |  |  |  |  |  |  |  |  |  |  |
| **A** | **1** | **A** | **2** | 0 | 0 | 0 | 21 | 6 | 12 | 121 | 2 | 0 | 41 |
|  |  | **B** | **1** | 3 | 0 | 0 | 23 | 8 | 17 | 126 | 0 | 0 | 26 |
|  |  | **C** | **1** | 3 | 0 | 0 | 53 | 5 | 11 | 125 | 0 | 0 | 6 |
|  |  | **D** | **1** | 5 | 0 | 1 | 45 | 6 | 26 | 113 | 2 | 0 | 5 |
| **A** | **2** | **A** | **1** | 0 | 0 | 0 | 31 | 12 | 24 | 122 | 4 | 0 | 3 |
|  |  | **B** | **1** | 0 | 0 | 0 | 29 | 7 | 23 | 118 | 0 | 0 | 17 |
|  |  | **C** | **1** | 1 | 0 | 0 | 51 | 6 | 10 | 117 | 0 | 0 | 9 |
|  |  | **D** | **1** | 3 | 1 | 1 | 35 | 6 | 38 | 102 | 3 | 0 | 5 |
| **B** | **1** | **A** | **1** | 19 | 7 | 1 | 36 | 28 | 95 | 129 | 2 | 0 | 79 |
|  |  | **A** | **2** | 18 | 1 | 0 | 45 | 22 | 54 | 118 | 3 | 0 | 132 |
|  |  | **C** | **1** | 1 | 0 | 0 | 124 | 16 | 50 | 193 | 2 | 0 | 7 |
|  |  | **D** | **1** | 29 | 0 | 1 | 78 | 13 | 71 | 187 | 3 | 1 | 10 |
| **C** | **1** | **A** | **1** | 14 | 3 | 1 | 42 | 15 | 73 | 125 | 5 | 1 | 180 |
|  |  | **A** | **2** | 16 | 1 | 0 | 40 | 10 | 45 | 117 | 2 | 0 | 228 |
|  |  | **B** | **1** | 1 | 0 | 0 | 47 | 21 | 22 | 193 | 0 | 0 | 175 |
|  |  | **D** | **1** | 10 | 2 | 2 | 63 | 14 | 56 | 264 | 3 | 2 | 43 |
| **D** | **1** | **A** | **1** | 20 | 7 | 2 | 60 | 39 | 158 | 113 | 3 | 1 | 128 |
|  |  | **A** | **2** | 17 | 0 | 0 | 67 | 21 | 129 | 102 | 1 | 0 | 194 |
|  |  | **B** | **1** | 14 | 1 | 0 | 85 | 23 | 82 | 187 | 2 | 1 | 136 |
|  |  | **C** | **1** | 2 | 0 | 0 | 123 | 22 | 83 | 264 | 2 | 1 | 34 |

# SI Figures


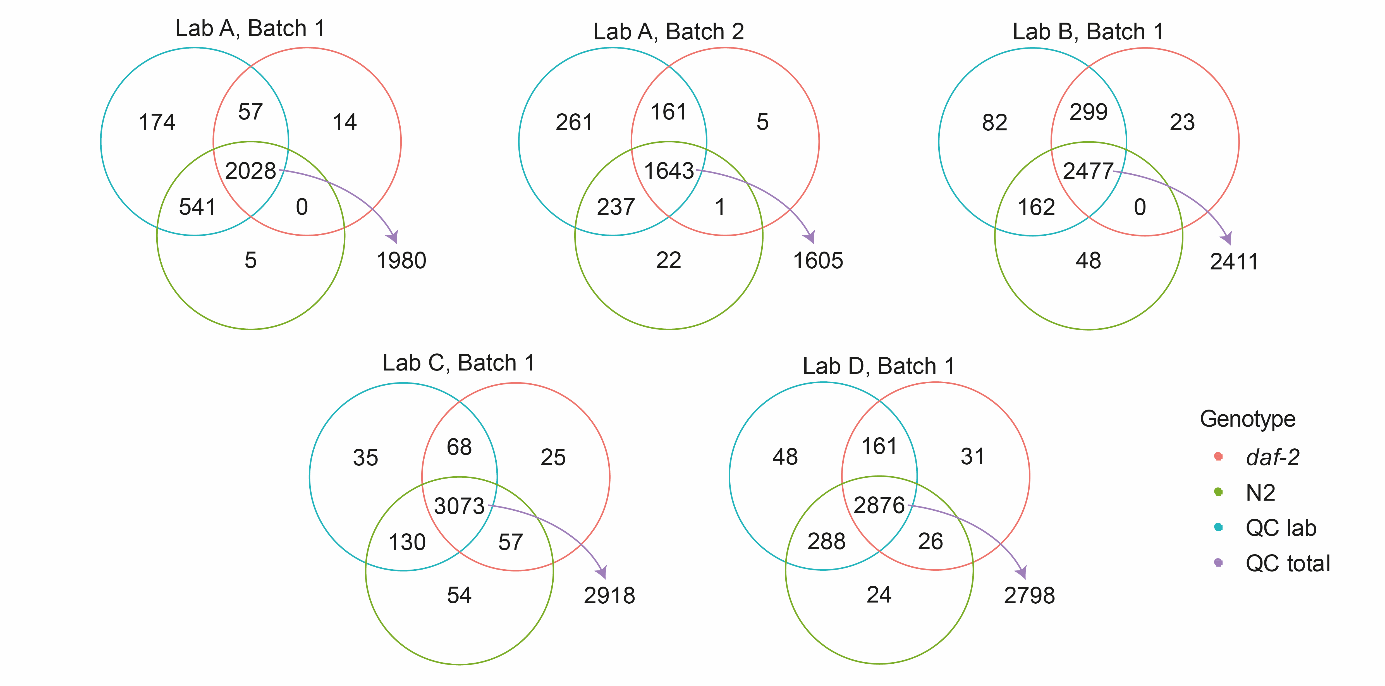


SI Figure 1: Visualization of filtering process. Each circle represents the number of features present in >80% of the respective group (N2, daf-2(e1370), laboratory/batch specific QC). The intersection is the number the fulfilled this criterion in all groups. Additionally, these features have been filtered to be present in >80% of the total QC and have an RSD < 30%. The numbers next to each Venn diagram represent the final number of features,


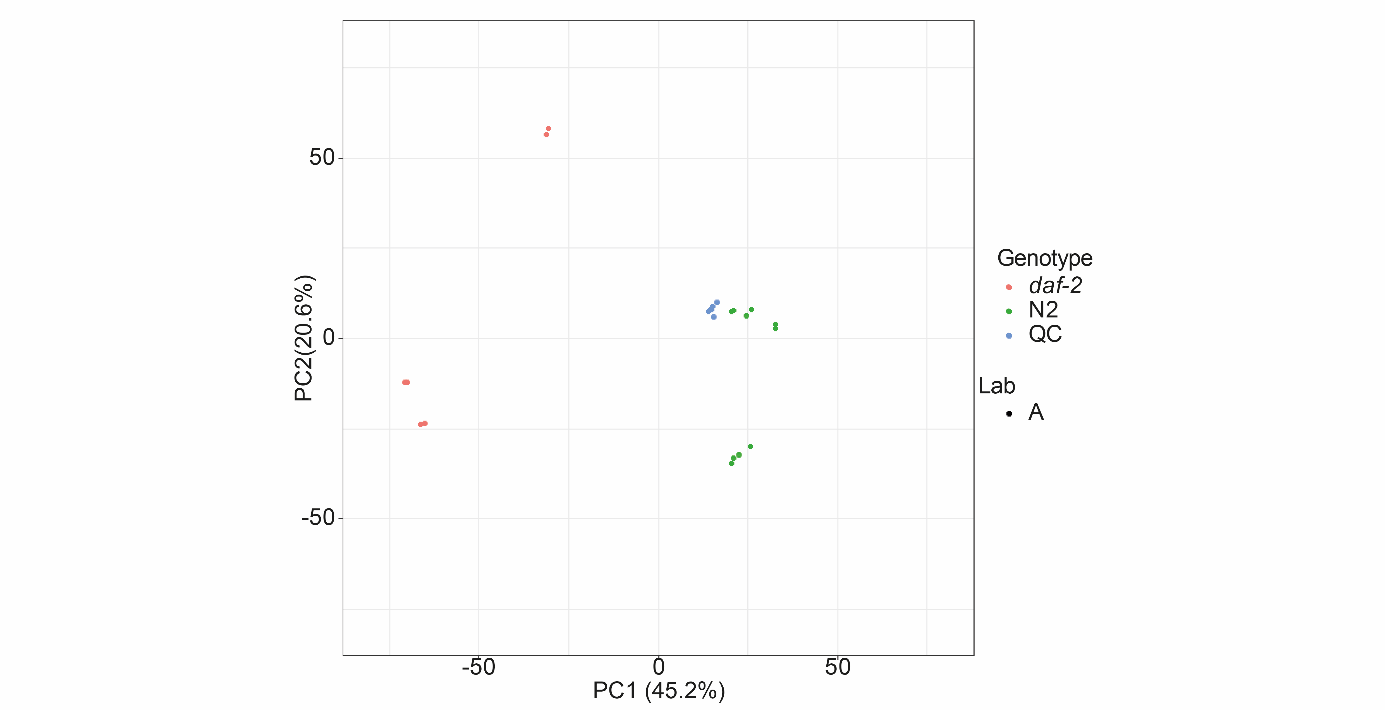


SI Figure 2: PCA scores plot of samples from laboratory A, batch 1.


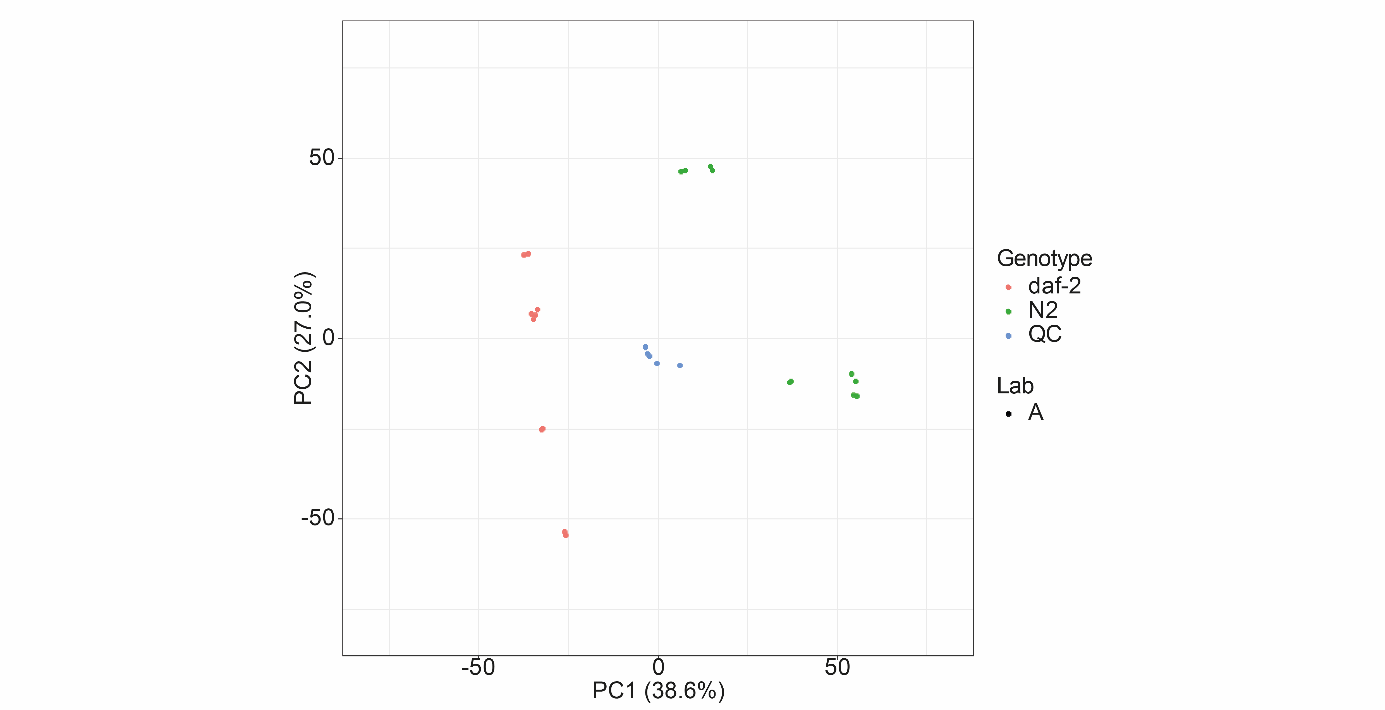


SI Figure 3: PCA scores plot of samples from laboratory A, batch 2.


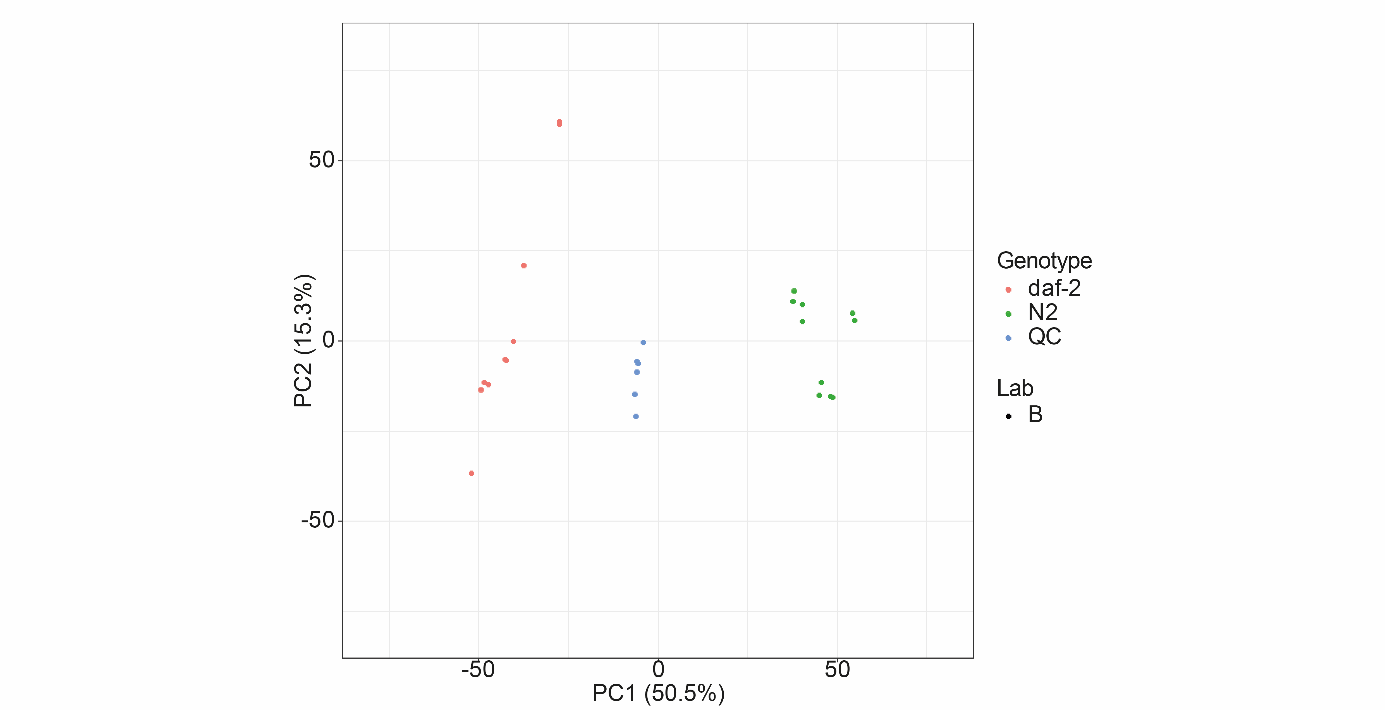


SI Figure 4: PCA scores plot of samples from laboratory B.


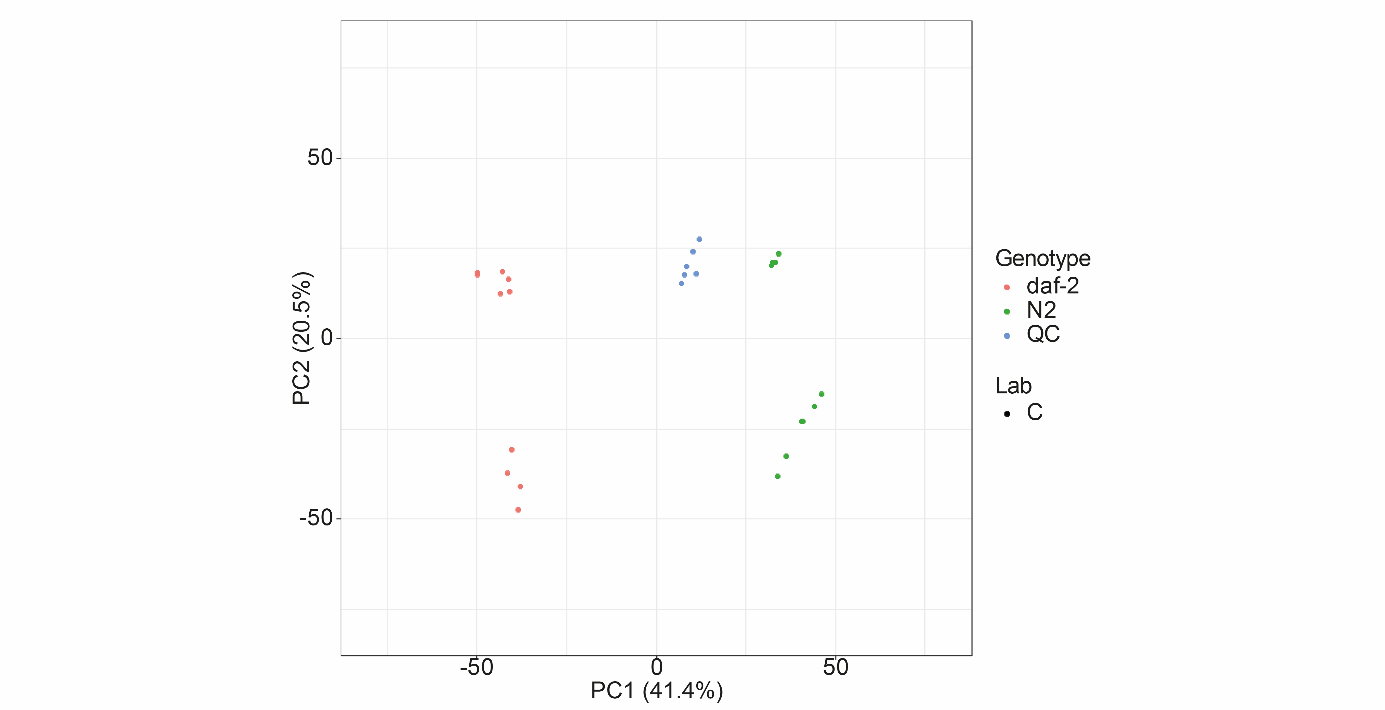


SI Figure 5: PCA scores plot of samples from laboratory C.


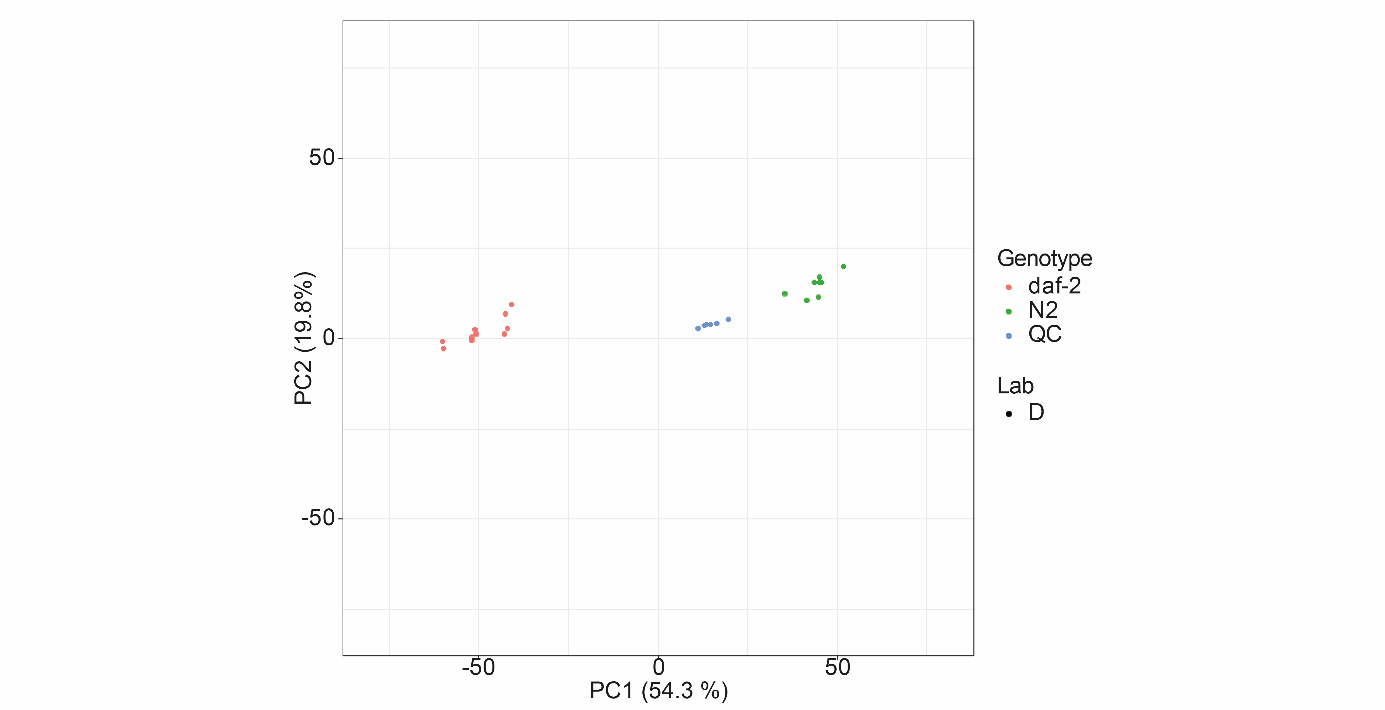


SI Figure 6: PCA scores plot of samples from laboratory D.


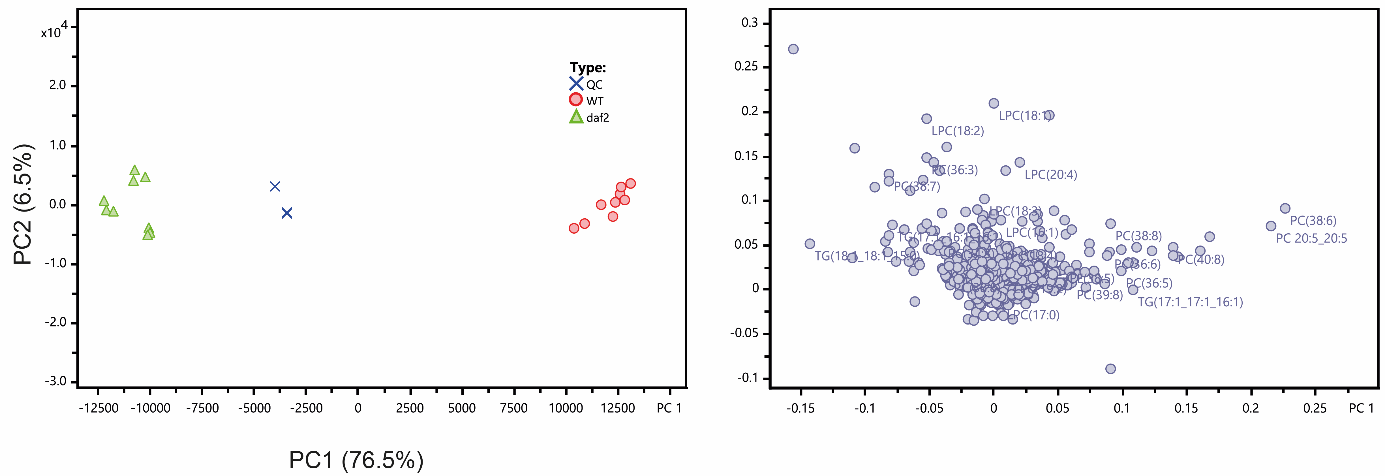


SI Figure 7: PCA scores and loadings plot from timsTOF data.
